# Regarding: “Hydroxychloroquine: a comprehensive review and its controversial role in coronavirus disease 2019”

**DOI:** 10.1080/07853890.2021.1872094

**Published:** 2021-01-13

**Authors:** Peter A. McCullough

**Affiliations:** Texas A & M University, College of Medicine, Professor (Affiliated), Department of Internal Medicine, Baylor University Medical Center, Dallas, TX, USA

The review article by Bansal and co-workers discusses hydroxychloroquine (HCQ), which at the moment is the most widely used prophylactic and therapeutic agent for SARS-CoV-2 infection (COVID-19) in the world [1]. The safety profile of this long-utilized antimalarial/anti-inflammatory is well understood and its benefit to risk relationship is encountered everyday as physicians prescribe this agent [2,3]. In Figure 4, the authors depict a large white scar in the short axis image of the left ventricle similar to that of a myocardial infarction. They also indicate that HCQ causes a cardiomyopathy. The references were reviewed and there is no support for large scars or cardiomyopathy after 5–30 days of HCQ use in acute COVID-19. Bansal states the cardiomyopathy is usually reversible and references Stein’s paper on neuromuscular findings in 10 cases found in 33 years, and none related to cardiomyopathy [4]. Recent monitored safety data on HCQ in critically ill patients with COVID-19 has been published in top tier journals and is consistent with its prior profile [5–7]. Bansal and co-workers’ graphic depiction of HCQ and myocardial scar in the figure is misleading to the readership and frightening to patients who rely on this medication when prescribed by competent physicians in the early multidrug treatment of COVID-19 to reduce the risk of hospitalization and death [8,9].
